# Health, wellbeing and lived experiences of adults with SMA: a scoping systematic review

**DOI:** 10.1186/s13023-020-1339-3

**Published:** 2020-03-12

**Authors:** Hamish W. Y. Wan, Kate A. Carey, Arlene D’Silva, Steve Vucic, Matthew C. Kiernan, Nadine A. Kasparian, Michelle A. Farrar

**Affiliations:** 1 Discipline of Paediatrics, School of Women’s and Children’s Health, UNSW Medicine, UNSW Sydney, Sydney, NSW 2031 Australia; 2 Department of Neurology, Westmead Hospital and Western Clinical School, University of Sydney, Sydney, Australia; 3 Brain & Mind Centre, University of Sydney, Institute of Clinical Neurosciences, Royal Prince Alfred Hospital, Sydney, NSW 2006 Australia; 4 Cincinnati Children’s Center for Heart Disease and the Developing Mind, Heart Institute and the Division of Behavioral Medicine & Clinical Psychology, Cincinnati Children’s Hospital, Cincinnati, OH USA; 5 Department of Pediatrics, University of Cincinnati College of Medicine, Cincinnati, OH USA; 6 Department of Neurology, Sydney Children’s Hospital, Randwick, NSW 2031 Australia

**Keywords:** Spinal muscular atrophy, Adult, Healthcare, Mental health, Natural history

## Abstract

**Background:**

Spinal muscular atrophy (SMA) is a neurodegenerative disease that has a substantial and multifaceted burden on affected adults. While advances in supportive care and therapies are rapidly reshaping the therapeutic environment, these efforts have largely centered on pediatric populations. Understanding the natural history, care pathways, and patient-reported outcomes associated with SMA in adulthood is critical to advancing health policy, practice and research across the disease spectrum. The aim of this study was to systematically review research investigating the healthcare, well-being and lived experiences of adults with SMA.

**Methods:**

In accordance with the Preferred Reported Items for Systematic Reviews and Meta-Analysis guidelines, seven electronic databases were systematically searched until January 2020 for studies examining clinical (physical health, natural history, treatment) and patient-reported (symptoms, physical function, mental health, quality of life, lived experiences) outcomes in adults with SMA. Study risk of bias and the level of evidence were assessed using validated tools.

**Results:**

Ninety-five articles met eligibility criteria with clinical and methodological diversity observed across studies. A heterogeneous clinical spectrum with variability in natural history was evident in adults, yet slow declines in motor function were reported when observational periods extended beyond 2 years. There remains no high quality evidence of an efficacious drug treatment for adults. Limitations in mobility and daily activities associated with deteriorating physical health were commonly reported, alongside emotional difficulties, fatigue and a perceived lack of societal support, however there was no evidence regarding effective interventions.

**Conclusions:**

This systematic review identifies the many uncertainties regarding best clinical practice, treatment response, and long-term outcomes for adults with SMA. This comprehensive identification of the current gaps in knowledge is essential to guide future clinical research, best practice care, and advance health policy with the ultimate aim of reducing the burden associated with adult SMA.

## Background

Management of SMA in adulthood has traditionally been limited to long-term multidisciplinary medical and supportive care to maintain functional mobility, independence and quality of life [1–3], favorably altering natural history [4]. Since 2017, there have been major transformations in medical therapy for pediatric SMA, including two Federal Drug Administration approved disease-modifying treatments [5–8]. Notwithstanding these milestones, there remain many uncertainties regarding treatment response, effects and long-term outcomes across this diverse clinical population, particularly for adults with SMA [9].

Clinically, SMA is characterized by progressive muscle weakness and atrophy due to degeneration of motor neurons of the spinal cord and cranial motor nuclei [2]. This neurodegeneration is caused by a homozygous deletion or mutation of the “Survival of Motor Neuron 1” (*SMN1*) gene [10, 11], with a pan-ethnic incidence of 1 per 11,000 live births and a prevalence of around 1–2 per 100,000 persons [12, 13]. The clinical phenotype has a broad spectrum of severity which is classified based on age of onset and maximal motor milestone achieved: people with SMA type I have symptom onset within 6 months and never attain independent sitting; people with SMA type II have onset between 6 and 18 months and achieve unassisted sitting but not independent walking; people with SMA type III have onset between 18 months-18 years and attain the ability to walk unaided; people with SMA type IV have onset > 18 years.

The health of adults diagnosed with SMA extends from the physical impact to their mental and social well-being, highlighting the importance of a comprehensive and interdisciplinary approach for clinical care and research [1, 3, 14]. Although it is estimated that adults comprise more than 25% of the global SMA patient population [15], reports have documented differences in clinical and supportive care, as well as challenges in accessing new therapies and research [16, 17]. Thus, the purpose of this scoping review was to synthesize and critically appraise the available evidence on the natural history, clinical management, physical and mental health, quality of life, and lived experiences of adults with SMA to advance future health policy, practice and research.

## Methods

### Search strategy and selection criteria

A scoping review systematically charts diverse bodies of evidence to identify key concepts and gaps in research [18]. The Preferred Reporting Items for Systematic reviews and Meta-Analyses (PRISMA) guidelines were followed to identify and screen scientific literature published in English, and extract data [19]. A comprehensive search was performed using seven electronic databases (Medline, PsycINFO, Scopus, Embase, CINAHL, ProQuest, Cochrane) from inception to January 20 2020 and limited to human studies. Ancestry methods, citation chaining and prolific author searching were used to identify additional articles up until the point of manuscript submission. Search terms included “spinal muscular atrophy”, “adult”, “adolescent”, “patient”, “family”, “lived experience”, “quality of life”, “patient perspective”, “mental health”, “adaptation, psychological”, “natural history”, “treatment”, “care”, “activities of daily living”, “living conditions” and “patient experiences”.

Studies involving adults (for the purposes of this review, adult was considered anyone over the age of 17 years) diagnosed with SMA were included for review if they described the (i) clinical characteristics or natural history of SMA; (ii) clinical management or treatment; or (iii) perspectives of adults with SMA regarding their health and well-being or lived experiences. Studies with mixed samples where outcomes for adults with SMA were not reported separately or could not be calculated, non-peer reviewed articles, and publications in a non-English language were excluded. After removal of duplicates, two reviewers (HW, KC) independently screened titles and abstracts for eligibility. The full-text of studies assessed as potentially relevant were independently evaluated by two reviewers (HW, KC). Differences in evaluation were resolved through consensus or consultation with a third reviewer (MF).

### Data extraction

Data extraction was carried out by one of three authors (HW, KC, AD) using a standardized data collection form, and checked for accuracy and completeness by a second author. Extracted data included study and sample characteristics, intervention and comparator details, outcomes measured and results, as relevant to the current review.

### Assessment of bias and quality of evidence

Eligible original research articles (excluding case series or case studies) were rated using the QualSyst tool independently by two reviewers (HW, KC, and/or AD) [20]. QualSyst is a standardized, reproducible and quantitative means of simultaneously assessing the quality of research, suitable for use with a broad range of study designs (quantitative and qualitative). Quality is defined in terms of the internal validity of studies, or extent to which the design, conduct and analyses minimize errors and biases, yielding a score between 0 and 1 for each study, with higher scores indicating lower risk of bias and thus greater methodological rigor (> 0.8 = ‘Strong’, 0.71–0.79 = ‘Good’, 0.50–0.70 = ‘Adequate’, < 0.50 = ‘Limited’). The QualSyst score was not used to limit articles for the review. In addition, for each of the main areas of clinical management [1, 3], ratings of the level of evidence were undertaken utilizing the Oxford Centre for Evidence-based Medicine for ratings of individual studies [21].

### Data synthesis and analysis

The studies were categorized based on reporting clinical (disease manifestation, natural history, management) or patient-reported outcomes (physical symptoms and function, mental health and psychosocial well-being and social participation) for adults with SMA. The primary reporting methods was narrative synthesis. Statistical synthesis using meta-analysis was not considered informative, given the small number of studies for each category and disparate methods, outcome measures, and study designs.

## Results

**Fig. 1:**
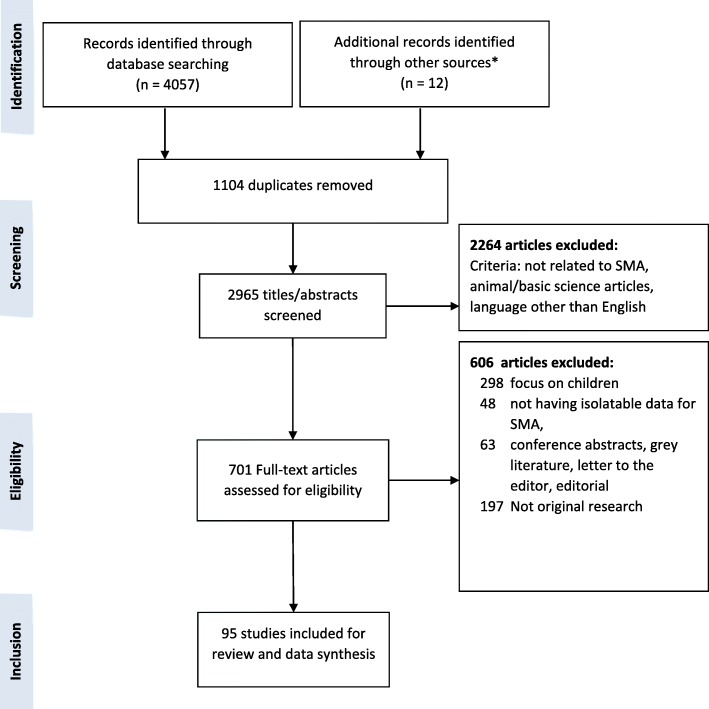
Study flow chart. *Additional references identified through other sources included manual searches of reference lists of identified studies and review articles, and prolific author searches

The search of databases, citations and reference lists yielded 4057 records. Following removal of duplicates (*n* = 1104), 2965 titles and abstracts were screened, and the full-text of 701 articles reviewed with 95 articles meeting all eligibility criteria (see Fig. 1). This comprised 62 quantitative, 9 qualitative, 4 mixed methods studies, 16 case series/reports, and 4 consensus statements on standards of care.

### Clinical characteristics of SMA in adulthood

Physical symptoms for adults with SMA included limitations in muscle strength, mobility and posture, and respiratory and bulbar function [2, 22–27]. The magnitude of these limitations varied among adults with different SMA types [27]. Hip dislocation or subluxation has been reported in approximately 33% of people with SMA type II or III in long-term follow-up [28].

### Natural history

Of the 19 studies that examined the natural history of SMA during adulthood (Table 1), muscle strength or motor function was the primary outcome in 14 studies. Comparisons of muscle strength and motor function between younger (children and adolescents) and older (adult) individuals with the same SMA phenotype in cross-sectional studies demonstrated significant differences in muscle strength and loss of motor skills over time [22, 30, 32, 36, 39, 41, 42, 45–47]. The estimated rate of muscle strength decline was determined in one cross-sectional study, with mean annual losses of 1 point for the summative Medical Research Council (MRC) score and 0.5 for the Hammersmith Functional Motor Scale Expanded (HFMSE) [22]. Five studies have assessed ambulatory status with the probability of continued ambulation at 20 years of age ranging between 16 and 33.5% for SMA type IIIa and 84–100% for SMA IIIb [29, 30, 32]. Whilst the probability of ambulation either after age 40 years or 40 years post onset ranged from 22 to 45% for SMA type IIIa (onset < 3 years) and 50–68% for SMA type IIIb (onset > 3 years) [29–31, 41]. Variability in the results of prospective longitudinal studies measuring motor function and/or strength were evident with two studies showing no change [33, 37], while others demonstrated gradual decline, particularly when observational periods extended beyond 2 years [26, 34–36, 38–43]. Separately, functional decline was not solely related to muscle weakness, with factors such as disuse atrophy, contractures and co-morbidities identified as contributing to loss of function [42, 48–50]. Considerable differences between study designs (9 prospective, 7 retrospective, and 3 cross-sectional), participant characteristics (*N*:10–240, types of SMA, disease duration), primary outcome (survival, strength or motor function), length of follow up (0–17 years) and documentation of comorbidities limited the analysis of study findings for differences in the rate of deterioration of muscle strength between SMA types with age.

**Table 1:** Studies providing insights into natural history of adult SMA*

Source	Study design	Population (SMA type^**a**^, age^**b**^, number)	Primary outcome measure	Key findings	QUALSYST Score
Zerres et al., 1997 [29]	Multicentre retrospective study	SMA types II-III, *n* = 569	Survival probability, ambulatory probability	Survival rate for SMA II 68.5% at 25 years; SMA III not significantly different to normal population. Probability of ambulation at 40 years after onset 22% (SMA IIIa) and 58.7% (SMA IIIb)	0.86
Zerres & Rudnik-Schӧneborn, 1995 [30]	Multicentre, retrospective study	SMA type I-IV, *n* = 445 (197 SMA type I; 104 SMA II; 73 SMA IIIa; 61 SMA IIIb; 10 SMA IV)	Survival probability (types I and II), ambulatory probability (type III)	Survival probability at 20 years of age: 0 and 77% for type I and II respectively. Ambulatory probabilities at 40 years of age: 45 and 67% for type IIIa and IIIb respectively.	0.86
Chung Wong & Ip, 2004 [31]	Single centre retrospective study	SMA I-III, *n* = 83	Survival pattern, ambulatory status	Survival probabilities at 20 years: type I 30%; type II 92%; type III 100%) SMA IIIa and IIIb have a 38 and 68% chance of remaining ambulatory 40 years post disease onset.	0.82
Farrar et al., 2013 [32]	Single centre retrospective study	SMA I-III, *n* = 70 (20 SMA I; 31 SMA II; 14 SMA IIIa, 5 SMA IIIb)	Survival, ambulatory status	40 year survival probability of 0, 52 and 100% for SMA type I, II and III respectively. Ambulatory probability at 20 years of age = 16% for type IIIa and 100% type IIIb	0.91
Piepers et al., 2008 [33]	Multicentre, prospective study, follow up 30 months	SMA IIIb and IV, median age disease onset 22.2 years (range 10–37 years), *n* = 12	Muscle strength (MRC grading), respiratory function (FVC), QoL (SF-36)	No significant changes in outcome measures after 2.5 years follow up	0.70
Kaufmann P, et al., 2012 [34]	Multicenter, prospective study, average follow up 25 months	SMA II and III, mean baseline age 11.3 ± 9.4 years, *n* = 85 (41 SMA II, 38 SMA III)	Motor function (HFMS, HFMSE, GMFM), respiratory function (FVC), QoL (PedsQL), muscle strength (myometry)	Decline in motor function (−1.71 HFMSE; −4.39 GMFM; − 1.26 HFM) and respiratory function (− 3%) over time when evaluated beyond 12 months	0.95
Pera et al.*,* 2019 [35]	Muticentre longitudinal study over 12 months	SMA II and III, total cohort age range 2.7–49.7 years, *n* = 114. (*n* = 27 aged > 15 years)	Motor function (RULM)	RULM changes over 12 months − 0.6 (2.35SD), SMA II: 0.2 (1.8SD, *n* = 14); non-ambulant SMA III − 1.7 (2.4SD, *n* = 6); ambulant SMA III − 1.4 (2.7SD)	1.00
Wadman et al., 2018 [22]	Single centre cross-sectional study	SMA I-IV, age range = 1–77.5 years (60% ≥18 years), *n* = 180	Motor function (HFMS, HFMSE), muscle strength (MRC)	Progressive loss of muscle strength and function. Average decline was 1 MRC point and 0.5 HFMSE points per year.	0.91
Mercuri et al., 2016 [36]	Multicentre retrospective study with 12 month observation period	SMA II and III, baseline age = 2.5–55.5 years, mean age = 10.65 years, *n* = 268	Motor function (HFMSE)	Ambulant: 12 month change not associated with age Non-ambulant: 12 month change different among various age groups with slow functional loss (− 0.93 points/year) after age 15 years	0.95
Sivo et al., 2015 [37]	Single centre longitudinal study over 12 month	SMA II and III, age = 3.5–29.0 years (mea*n* = 10.22) *n* = 74 (SMA II = 70, SMA III = 4)	Motor Function (ULM, HFMSE)	The mean 12 month changes in the 9 patients > 18 years was 0.11 (range − 1 to + 1) for HFMSE and 0 (range − 1 to + 1) for ULM	0.82
Werlauff, Vissing & Steffensen 2012 [38]	Single centre, longitudinal prospective study, median follow up = 17 years	SMA II and III, baseline age = 6–53, *n* = 30 (23 SMA II, 7 SMA III)	Motor function (Brooke upper limb scale, EK), muscle strength (MMT)	Upper limb muscle strength declines slowly over time, but can only be detected if monitored over several years.	0.90
Montes et al., 2018 [39]	Multicentre longitudinal study, follow up 0.5–9 years	SMA IIIa and IIIb, *n* = 15 ≥ 20 years	Motor function (6MWT)	Loss of function evident with 6MWT mean annual rate of change − 9.7 m/year	0.82
Vuillerot et al., 2013 [40]	Multicentre, retrospective study, follow up over 1.2–66 months	SMA II (n = 44, mean age 11.5 ± 5.0, range 5.7–27); SMA III (*n* = 59, mean age 18.7 ± 12.3, range 6.2–59),	Motor function (MFM-32)	SMA II follow up > 6 months: − 0.9 points/year SMA III follow up > 6 months: − 0.6 points/year	0.77
Russman et al., 1996 [41]	Multicentre prospective study, 2 year follow up	SMA types II and III, *n* = 159 (40 patients > 16 years)	Motor function, ability to sit or walk	Slow loss of function, primarily related to maximum function achieved and age of onset. 50% of SMA III with onset aged 2–6 years lost walking ability by age 44 years.	0.70
Iannaccone et al., 2000 [42]	Longitudinal study, 2–6 year follow up	SMA type not specified, n = 30 aged ≥15 years (mean 30.3 ± 11.2 years)	Muscle strength (TMS in kg through myometry) and motor function	Patients age > 15 years had a mean change of − 0.4 in TMS	0.86
Carter et al., 1995 [43]	Single centre, prospective study, 10 year follow up	SMA II (mean age 17 ± 14 years) and III (mean age 40 ± 20 years), *n* = 45	Muscle strength (MMT)	Mean decline in combined MMT score per decade: SMA II = -0.24, SMA III = not significant	0.78
Durmus et al., 2017 [44]	Single centre cross-sectional study	SMA IIIb, mean age = 23.52 years (13–48), *n* = 25	Muscle MRI, muscle strength (MRC)	Significant correlation of loss in muscle strength with time in iliopsoas and triceps but not in other muscles.	0.70
Deymeer, 2008 [26]	Single centre prospective study, ≥10 year follow up	SMA IIIb, median age of onset 12.5 years (range 9–18 years), n = 10	Muscle strength (MRC)	Decline usually ≤ 1 MRC grade for each 5-year period in each muscle group, with consistent patterns amongst each patient. Triceps, iliopsoas, thigh adductors and quadriceps femoris preferentially affected	0.67

Abbreviations: *6MWT* Six-minute walk test, *EK* Egen Klassifikation, *FVC* Forced vital capacity, *GMFM* Gross Motor Function Measure, *HFMS* Hammersmith Functional Motor Scale, *HFMSE* Expanded Hammersmith Functional Motor Scale, *MFM* Motor Function Measure, *MMT* Manual muscle testing, *MRC* Medical Research Council, *QoL* Quality of Life, *RULM* Revised Upper Limb Module, *TMS* Total Muscle Score, *ULM* Upper Limb Module

^a^ Traditional SMA classifications: Type I - symptom onset < 6 months, unable to sit independently; Type II - symptom onset between 6 and 18 months, achieved ability to sit independently; Type IIIa - symptom onset < 3 years, achieved ability to walk independently; Type IIIb – symptom onset > 3 years, achieved ability to walk independently; Type IV - adult onset SMA

^b^The data presented summarizes salient findings relevant to the natural history of adults with SMA, results pertinent to childhood trajectories have been omitted

^c^ Independently rated by at least two of the study authors using the QUALSYST assessment tool with higher scores indicating lower risk of bias and thus greater methodological rigour (> 0.8 = ‘Strong’, 0.71–.0.79 = ‘Good’, 0.50–0.70 = ‘Adequate’; < 0.50 = ‘Limited’)

### Clinical management

Forty-six of the 95 captured articles examined clinical care of SMA in adults. Although the established standards of care for SMA have primarily focused on children with severe phenotypes [1, 36], clinical management for adults with SMA remains multidisciplinary, ideally in a specialized services incorporating nutritional, respiratory, orthopedic, and rehabilitation care. Four studies reported feeding and swallowing difficulties resulting from bulbar weakness in adults with SMA. Difficulties included reduced bite force, increased masticatory muscle fatigue and difficulties with mouth opening, together prolonging meal times [51] and adversely impacting nutritional intake [27, 52, 53]. Conversely, in a cohort of non-ambulatory, high-functioning people with SMA, 71% had a fat max index (fat mass/height^2^, estimated using dual energy X-ray absorptiometry) >85th percentile for age and gender compared to 47% of low-functioning, non-ambulatory and 47% of ambulatory SMA patients [54]. Six studies measured respiratory function in 321 adults with SMA and showed slow and progressive decreases in respiratory capacity [55–57] and earlier, more rapid progression of respiratory dysfunction in adults with SMA type II [34, 58]. Stratification of SMA III by subtype suggested respiratory function in type IIIa SMA was similar to SMA type II whilst respiratory function in type IIIb and IV patients was comparable to age-matched healthy controls [33, 57]. Despite this and consensus on recommendations for the monitoring and initiation of respiratory support [1, 59], respiratory management remains variable [17, 60]. Recommendations for rehabilitation therapy such as physical therapy were described in the 2018 standards of care' [3], yet the effect of these recent guidelines in the care of people with SMA is not yet known. In an earlier study, half of adults (*n* = 19, United States) reported receiving only one physical therapy service on average per month [61], and there was no literature evaluating the impact of such services. Case reports described development of assistive technology trialed by 11 adults with SMA and severe motor disabilities, which included an upper limb assistive exoskeleton, eye tracking system and computer assisted robot, with enhanced functionality in self-feeding and communication reported [62–65].

### Medications

Five randomized placebo controlled clinical trials and three open label trials evaluated medications (valproic acid, gabapentin, salbutamol, hydroxyurea and nusinersen) in adults with SMA type II and III (*n* = 362 participants, follow up 6–12 months), using different measures of muscle strength and function: four showed no statistically significant effects on the outcome measures, and an open label study of nusinersen showed mean improvement of 8.25 m in the 6 min walk test (*p* = 0.01), Taken together these were rated as providing Level 1–4 evidence (Table 2) on the Oxford Centre for Evidence-based Medicine scale. Seven case series reported administration of intrathecal nusinersen, with radiological guidance an option for administration, using transforaminal or interlaminar approaches +/− laminotomy [89–95]. Lumbar punctures were mostly well tolerated, adverse events included post lumbar headache and subarachnoid haemorrhage.

**Table 2:** Management approaches commonly used for adults with SMA

**Clinical Issue**	**Assessments and monitoring**	**Management and sources of evidence**	**Level of evidence**^**a**^	**Context for clinical implementation**	**References**
**Rehabilitation**					
Muscle weakness limiting mobility, function and activities of daily living	Optimal methods for evaluation and monitoring for adults are lacking	Assistive devices Consensus statements and surveys describing use of orthoses and assistive devices	Level V	Limited evidence for selection of optimal assistive devices	[3, 27, 60, 66]
		Qualitative studies incorporating patient experience	Unclear – patient reported benefits		[67–70]
		Case reports of development and trial of upper limb assistive exoskeletons	Level V		[63, 64]
		Pilot study of a brain computer interface	Level V		[62]
		Case report of ocular movement detector system to aid communication in severe SMA	Level V		[65]
		Exercise Single randomised controlled trial in ambulatory SMA of 14 patients – no change in 6MWT, fatigue or function, improvement in V0_2_max.	Level III	Further evidence is needed to develop exercise guidelines	[71]
		Open label study in 6 SMA III patients – training improves oxidative capacity, induces fatigue	Level IV		[72]
**Respiratory Care**					
Impaired cough and ability to clear airway sections	Evaluate cough effectiveness	Reviews and consensus statements outlining methods for airway clearance; case series of NIV, manually assisted coughing, MI-E	Level IV	Evidence supporting optimal methods for evaluation and management are lacking	[1, 59, 73]
Respiratory muscle weakness	Monitor gas exchange for evidence of hypoventilation	Respiratory support for chronic ventilatory insufficiency: Non- invasive ventilation Consensus statements, surveys, observational study and case reports describing use of non-invasive ventilation	Level V		[1, 17, 59, 60, 74–76]
		Invasive ventilation Case studies and surveys describing use of invasive ventilation in a small number of adults	Level IV	Invasive ventilation when NIV is insufficient is an individual decision incorporating views of person and quality of life	[77] [60]
Recurrent respiratory infections		Immunizations Consensus statements	Level V		[1, 59]
**Gastrointestinal and Nutritional Care**					
Feeding and swallowing difficulties associated with lower body weight and increased risk of aspiration pneumonia	Speech therapist and dietitian evaluation of feeding, swallowing and nutrition	Reviews and consensus statements describe modifying food consistency, optimizing oral intake, enhance feeding with positioning, seating and equipment Gastrostomy to provide nutritional supplementation when oral intake inadequate	Level V	No evidence to support specific diets. No consensus on when to commence enteral supplementation: an individual decision incorporating views of person and quality of life	[3]
		Surveys and case reports describing frequency and management of feeding difficulties in adult SMA	Level IV		[78, 79] [52]
Gastrointestinal dysmotility (reflux, constipation and delayed gastric emptying)		Consensus statements suggests gastroesophageal reflux medications, prokinetic agents, aperients, nissen fundoplication	Level V		[3]
**Disease modifying medication**					
**Agent**	**Study design**	**Results**	**Level of evidence**^**a**^	**Context for clinical implementation**	**References**
Valproate	Prospective randomized placebo controlled cross-over trial of 33 ambulatory adults with SMA	No change in max voluntary isometric contraction, pulmonary, electrophysiological, or functional outcomes	Level I	Valproate is well tolerated, no improvement in strength or function in adults with SMA	[80]
	Open label, 6 adults with SMA II and III Retrospective open label, 7 adults with SMA III and IV, mean duration 8 months	No change in motor function, variable changes in pulmonary outcomes Increased quantitative muscle strength and subjective function	Level V Level V		[81] [82]
Gabapentin	Randomized controlled vs no treatment in 120 adults with SMA II and III over 12 months	Improvement in leg muscle strength at 12 months; no change in functional tests or FVC.	Level IV	Inconsistent evidence of efficacy	[83]
	Randomized double blind placebo controlled trial in 84 adults with SMA II and III	No differences between placebo and drug in strength, FVC, functional rating scale, impact profile	Level I		[84]
Hydroxyurea	Randomized, double-blind, placebo controlled trial – 55 SMA II and SMA III patients (aged 5–41)	No improvements in motor or respiratory function, increased development of neutropenia in the hydroxyurea group	Level I	No evidence of efficacy	[85]
Salbutamol	Randomized, double-blind, placebo-controlled trial – 45 SMA III (aged 21–53 years), 12 months duration Open label, 10 patients (aged 28–61 years), 12–72 months of salbutamol treatment	Safe and well tolerated. Significant and progressive increase in blood SMN2 full length protein in peripheral blood in salbutamol treated patients. Patients reported benefits of decreased fatigue, improved functioning, infrequent side effects	Level II Level IV	Underpowered to demonstrate clinical efficacy Unclear – patient reported benefits	[86] [87]
Nusinersen	Prospective open label observational study, 19 SMA III (aged 18–59 years), 10 months duration Retrospective open label case series, 4 adults SMA III (aged 19–52 years), follow up 4–20 months Descriptive studies of repeated intrathecal nusinersen administration in adolescent and adult patients – 78 patients in total (aged 11–61 years)	Statistically significant change in 6MWT (mean improvement 8.25 m), RULM and peak cough flow with negligible effect size. Well tolerated, adverse events of back pain in 7 and post lumbar puncture headache in 4 patients. Stable RULM, subjective improvement in endurance, hand strength, bulbar functioning Adverse events: headache, back pain CT guidance an option for administration using transforaminal or interlaminar approaches +/− laminotomy. Lumbar punctures were mostly well tolerated; adverse events included post lumbar headache and subarachnoid haemorrhage in 1 patient.	Level III Level IV Level IV	Mild treatment effect in adults with chronic SMA Available outcome measures not adequate to capture meaningful subjective improvements Feasibility and safety of intrathecal treatment with nusinersen demonstrated in adolescent adult patients with SMA II and III. Treatment can be medically and logistically challenging due to the clinical features of SMA.	[88] [89] [90–95]

^a^ Level of evidence according to the Oxford Centre for Evidence-based Medicine: Level I - Properly powered and conducted randomised clinical trial; systematic review with meta-analysis, Level II - Well-designed controlled trial without randomization; prospective comparative cohort trial, Level III - Case-control studies, retrospective cohort study, Level IV - case series with or without intervention; cross-sectional study, Level V - Opinion of respected authorities; case reports

Abbreviations: *MI-E* Mechanical insufflation-exsufflation, *FVC* Forced vital capacity, *VO*_*2*_*Max* Maximal oxygen uptake;

There was no literature that described mental health care for adults with SMA or evaluated transition into the adult service. Palliative care services for adults with SMA have been described in two studies; however, limited evidence suggests a lack of co-ordination in the provision of such care [96, 97]. Routine cardiac surveillance was not endorsed; two studies suggested that cardiac abnormalities were uncommon in type in SMA II and III patients [98, 99]. Four case series reported successful pregnancies among 39 adults with SMA [100–103]. One of these compared the incidences of obstetric complications to those in the normal population, and found statistically significant increased rates for preterm deliveries (29.4%) and caesarean sections (42%) [100].

### Lived experiences and patient-reported outcomes associated with SMA in adults

Twenty one of the 95 captured studies investigated the experiences of adults with SMA; 13 studies used a quantitative methodology (*n =* 1491), 6 used a qualitative methodology (*n =* 102), and 2 used mixed methods (*n =* 111).

### Physical symptoms and function

Physical health and functioning were measured in 3 studies using validated patient-reported quality of life measures (e.g. Short Form Health Survey, RAND-36) and all reported significantly lower ratings of health-related quality of life in the physical domain compared to published healthy reference populations [61, 104, 105]. Physical quality of life (QoL) significantly correlated with disease severity. Adults with SMA perceived limitations in physical functioning and ability to perform daily actions, especially independence in self-feeding, using the restroom and performing transfers, had the greatest impact on general well-being [27, 60, 67–69, 104–108]. One mixed methods study reported that women tended to prioritize the importance of certain activities (e.g. personal hygiene, eating) more than men, whilst the ability to utilize one’s hands and fingers was perceived as important by both genders [68]. People with SMA universally stated that maintaining stability of their current functional ability was important [60, 109]. One example was that small changes in being able to move one’s finger half an inch could be the difference between being able to operate a power wheelchair or not [109]. People with SMA described strategies to minimize the impact of mobility limitations, including the use of assistive devices, conserving energy for specific tasks, maximizing utility, pro-active health management, nutrition optimization, and minimizing exposure to infection [70]. Devices which improved independence, such as power wheelchairs and upper or lower limb orthoses, were highly valued [66].

Frequency and severity of bodily pain was measured in three questionnaires: in two customized surveys prevalence was 50–70% and rated as having a mild to moderate effect on life, compared to two studies using validated the short form health survey that reported no difference in bodily pain scores against the general population [27, 106, 110, 111]. Bodily pain was moderately associated with global ratings of life satisfaction [110].

### Psychosocial wellbeing

Mental health and psychosocial functioning were measured in adults with SMA in 9 studies (5 undertaken in The Netherlands) using 10 different validated patient report measures (e.g. Short form Health Survey, RAND 36, NMS Quest) and two customized surveys) [27, 104, 105, 108, 111–114]. Compared to published healthy age-matched reference populations, ratings of health-related quality of life in the mental domain were higher or similar [104, 105, 108], and ratings of psychological well-being, self-esteem, satisfaction with life and emotional functioning were similar. Subjective feelings of depression and anxiety were low in adults with SMA [112], yet correlated with feelings of fatigue [111]. Three of the 19 studies found an association between less severe physical limitations, for example ambulation, and more emotional distress [105, 111, 113]. Perceptions of fatigue were universal and not associated with physical function, health related quality of life or fatigability in another study [115]. For adults with poorer physical function, engagement with a patients’ association was associated with greater psychosocial wellbeing [114]. In a recent survey of 82 adults with SMA in the United Kingdom, 79% of participants agreed or strongly agreed that “people with SMA can live a fulfilling life” and 52% endorsed the statement, “having SMA causes people to suffer”. Only 29% of participants in this study perceived their health as ‘good’ [113]. Prevalence of non-specific “emotional issues” was 60% and average effect on daily life was rated as mild to moderate in a second customized questionnaire of 99 adults with SMA, with 80% also reporting impaired body image due to disease [27, 67].

The lived experience of 127 adults with SMA interviewed across 9 studies provides further insights into emotional and social well-being. (Table 3) The factors identified as important to psychological well-being included autonomy, competence and social participation along with resilience, determination, hope and an optimistic view of life [69, 70, 117]. The major emotional challenges voiced by people with SMA were coping with frustration, guilt and stress alongside desire for independence [67–69]. Fear of functional decline and premature death were also expressed in 6 studies, associated with the need to make difficult treatment decisions and readjust future hopes, expectations and goals [69, 70, 105, 116–118]. Physical decline following long periods of stability was associated with emotional distress [117, 118]. Participants in one study described unmet physical and psychological care needs in addition to difficulties associated with accessing appropriate adult healthcare services [117].

**Table 3:** Studies describing the lived experiences

Reference/ country	Study design	Sample	Key findings	QUALSYST score^**a**^
Ho et al*,* 2016 [69] Taiwan	Qualitative study Purposive sampling of cross-sectional cohort	Adults (Age = 25–54 years, mean 34.4) *N* = 9, (2 SMA II; 7 SMA III) Mandarin/Taiwanese speaking	Experienced a loss of control from declining muscular strength and independence. Utilised assistive devices and environmental manipulation to maximise function. Transcended limitations through striving to maximise independence and continue achieving key financial, educational and relational goals.	0.9
Lamb & Peden, 2008 [70] USA	Qualitative study Recruitment through patient support group	Adults (Age = 26+ years) *N* = 11 (4 SMA II; 7 SMA III) English speaking	People with SMA utilised creative and innovative methods for overcoming physical challenges. Maintaining strong relationships with family, friends and community and an optimistic life view was important.	0.8
Jeppesen et al., 2010 [68] Denmark	Cross-sectional mixed methods design, semi-structured survey with narrative inquiry	Adults ≥ 18 years *N* = 29 (All SMA II)	People with SMA faced multiple difficulties but managed to achieve large landmark “goals/achievements”; are in a state of striving to maintain optimism in spite of constant stressors.	0.79/0.75
Hunter et al., 2016 [67] USA	Qualitative study Purposive sampling	Adults (Age = 18–69 years, mean = 34) *N* = 15 (5 SMA II; 10 SMA III)	Most concerning issues included personal hygiene, dressing, walking and independence. Social and emotional issues included mainstreaming into society and self-confidence.	0.65
Qian et al., 2015 [116] USA	Qualitative study Purposive sampling	Children and adults with SMA (Age = 8–46 years, 33% were > 18 years). *N* = 21 (1 SMA I; 8 SMA II; 12 SMA III) Parents of people with SMA (*N* = 64)	SMA left a constant psychological threat of disease progression and premature death. High level of burden included social limitations, difficulties achieving independence, and financial pressures.	0.82
Rouault et al., 2017 [60] Countries across Europe	Cross-sectional survey Purposive sampling	People with SMA (*n* = 436; 2–65 years; 52% ≥ 19 years); Parents of people with SMA (*n* = 370); Other or unknown(*N* = 16)	Activities with the biggest impact on QoL were: ‘using the rest-room independently’, ‘self-feeding’, ‘turning in bed’, ‘washing’ and ‘transferring independently’.	0.87
Kruitwagen-van Reenen et al., 2018 [111] The Netherlands	Cross-sectional surveys Recruitment through patient support groups and clinics.	Adults with SMA (20–70 years); *n* = 62 (4 SMA I; 21 SMA II; 13 SMA IIIa; 20 SMA IIIb; 4 SMA IV)	People with early onset SMA experience more participation restrictions but similar levels of satisfaction compared with people with later onset SMA. Motor skills, feelings of depression and fatigue are correlates of participation in daily life.	0.93
Mongiovi et al., 2018 [27] 34 countries world-wide	Cross-sectional study Recruitment through the International SMA Patient Registry	Adults with SMA (18–81 years); *n* = 359 (16 SMA I; 132 SMA II; 144 SMA type III; 30 SMA IV; 34 unknown)	Limitations with mobility or walking, inability to do activities, weakness, and fatigue. Limitations with mobility had the greatest impact on the lives of adults with SMA.	0.87
Wan et al., 2019 [117] Australia	Qualitative study Purposive sampling	Adults and adolescents with SMA, parents and partners of people with SMA; n = 25 (19 people with SMA, 5 parents, 1 partner)	Participants report widespread unmet physical and mental healthcare needs, disengagement from adult healthcare services, as well as pride in resilience, personal accomplishments and social relationships.	0.93

Abbreviations *QoL* Quality of Life,

^a^ Independently rated by at least two of the study authors using the QUALSYST assessment tool with higher scores indicating lower risk of bias and thus greater methodological rigour (> 0.8 = ‘Strong’, 0.71–.0.79 = ‘Good’, 0.50–0.70 = ‘Adequate’; < 0.50 = ‘Limited’). For mixed methods studies, quantitative and qualitative components were assessed separately, and two summary scores were calculated (quantitative/qualitative)

### Experiences and participation in social activities

Adults with SMA perceived employment opportunities, economic independence and autonomy as key priorities [116], yet two thirds noted restrictions in work and education [111]. Six studies reported 33–66% of adults with SMA completed tertiary education [27, 68, 69, 108, 111, 119]. Full-time employment rates were reported to range from 18 to 49%, with most adults with SMA engaged in part-time employment [27, 68, 104, 108, 111, 119]. Reduced work hours have been associated with greater deterioration of motor function [119]. One-quarter to 61% of adults with SMA reported having a romantic partner or spouse; people with greater severity of symptoms were less likely to have a partner [68, 106, 108, 111, 119].

Socially, a majority of people with SMA reported experiencing stigma, challenges to integrating into mainstream society and participating in normal activities, with 62–72% of people reporting decreased satisfaction and 66–72% reporting restrictions and performance in social situations [27, 111]. People with early onset SMA (SMA I, II, and IIIa) reported significantly more participation restrictions compared to with people with late onset SMA (SMA IIIb and IV) [27, 111]. Some people with SMA have noted only feeling that their disease was an issue when there was a lack of facilities to allow them to engage with normal activities [118]. In one survey, only 15% of adults with SMA agreed with the question “people with SMA are well supported by society” [113]. Frequently environmental barriers presented limitations, including a lack of accessible bathrooms or wheelchair access [116]. Supportive family, friends and community members were identified as key facilitators of positive coping and engagement [69, 70]. Despite restrictions in daily activities, another study found adults with SMA were very satisfied with participation in social activities [108], and regression analyses demonstrated that 30–50% of variance in psychological well-being was explained by participant’s societal participation and satisfaction for basic psychological needs. Another study showed frequency of participation in daily life correlated with pain, feelings of depression and fatigue [111].

### Risk of bias and quality of evidence

Methodological rigor across 78% of studies was ‘good’ or ‘strong’, with a median QualSyst score of 0.79 (Range 0.36–1.00; interquartile range 0.14). Evidence related to clinical care of SMA in adults was rated as generally poor (Level 4 or 5) [21]. Synthesizing studies assessing patient experience or patient-reported outcomes, risk of selection bias was high often due to the recruitment strategy adopted (e.g., opt in) or participant characteristics (e.g., under-representation of people with lower levels of educational attainment), information bias was modest due to use of non-validated survey instruments in several studies and potential concerns with generalizability were evident with 6/20 studies undertaken in The Netherlands.

## Discussion

Adults living with SMA face multi-dimensional challenges which encompass physical, psychological, social, financial and practical domains. Significant barriers exist for adults with SMA, limiting engagement with health care, and access to therapeutic interventions that aim to maintain stability, promote function and enhance quality of life [109]. The dynamic and rapidly changing SMA therapeutic landscape in pediatric patients serves to emphasize the importance of providing high quality evidence-based healthcare to optimize translation in the large population of adults living with SMA. However, the care and support needs, disease progression, mental health and social well-being of adults living with SMA is largely understudied. This systematic review is required to address this knowledge gap by comprehensively reviewing the available scientific literature for the purposes of guiding and extending translational research to address uncertainties across the broader population of adults with SMA regarding clinical course, impact and therapeutic response, together with elucidation of optimal supportive care.

Access to and reimbursement of expensive therapies for rare diseases relies heavily on the demonstration of tangible benefits and the evaluation of cost-effectiveness. The considerable heterogeneity and slow disease progression in adults with SMA has contributed to the paucity of research and lack of efficacy in the limited number of trials to date. Clinical trials which could address this issue require long durations and robust clinical outcome measures, drawing attention to the need for biomarkers which are sensitive to changes in disease progression and/or response to disease modifying agents [120]. Unlike studies in infants and children with SMA, in which circulating neurofilaments are emerging as potential measures to assess disease progression and response to nusinersen treatment [121], no studies in adults with SMA have yielded a robust, reliable measurement [86, 88, 122–126]. A potential complexity in trying to detect elevated markers of axonal degradation in adults with SMA is again the slow rate of progression. The structure of such clinical trials also requires further understanding of pathophysiology in order to direct and justify continued research. Understanding the natural history of the various adult SMA subtypes is a key step in evaluating the efficacy and value of emerging disease modifying therapies. Whilst several natural history studies have been conducted to evaluate motor strength progression in adults [22, 26, 29, 34, 36, 38, 41], the phenotypic variability, slow disease progression and sensitivity of outcome measures make it hard to compare and draw definitive conclusions. Optimizing the effectiveness of outcome measures remains a key issue, particularly in light of the significant benefit in quality of life derived from small changes in function in adults with SMA [109].

While awaiting the emergence of future disease modifying therapies [8, 127], it is vital that practitioners are aware of the range of supportive care options available for adults, strengthened by the generation of high-quality evidence. It is essential for future consensus guidelines to include a specific focus on the management of adults with SMA. Functional classification of adult phenotypes, incorporating changes with disease progression and encompassing the adult spectrum of severity, may better describe appropriate care and support requirements (Table 4). Integrating transitional care in neuromuscular clinics and elucidation of best practices may reduce the potential for poorer objective health outcomes, health service use and loss to follow-up shown in other chronic health conditions associated with poorly managed transitions [128].

**Table 4:** Adult phenotypes of spinal muscular atrophy

Functional classification^a^	Non-sitters	Sitters	Walkers
Traditional classification^b^	Types I, II and some IIIa	Type II and some IIIa	Type IIIb, IV and some IIIa
Physical manifestations	Very severe weakness: -Quadriplegia -Weakness of face and bulbar muscles -Small movements of distal limb muscles -Areflexia	Very severe weakness: -Paraplegia -Distal arm movement -Areflexia	Pattern of weakness: Legs>arms Proximal>distal Reduced or absent reflexes in legs, may be normal in arms Calf hypertrophy No facial or bulbar weakness
Complications/ comorbidities	-Severe restrictive respiratory disease Ventilator support, Recurrent pneumonia/aspiration ±tracheostomy -Severe scoliosis /spinal fusion Contractures	Respiratory disease Non-invasive ventilation Scoliosis	Normal respiratory function

^a^ Functional classifications compiled from adult SMA descriptions in the literature [2, 22, 25–27]

^b^ Traditional SMA classifications: Type I - symptom onset < 6 months, unable to sit independently; Type II - symptom onset between 6 and 18 months, achieved ability to sit independently; Type IIIa - symptom onset < 3 years, achieved ability to walk independently; Type IIIb – symptom onset > 3 years, achieved ability to walk independently; Type IV - adult onset SMA

The experience, perceptions and involvement of adults with SMA is essential in future research, provision of health care and policy to drive more meaningful and patient centered outcomes. Studies incorporating the perspectives and experiences of adults with SMA are increasing our understanding of the impact of SMA on quality of life, mental health, emotional and social functioning. Clinically, periods of significant change in physical ability are associated with distress, however mental health does not have a linear association with severity of physical disability, indeed those with milder phenotypes have reported greater difficulties. Characterizing potential determinants of quality of life, emotional issues and deficiencies in social support are a first step toward developing evidence-based psychosocial care.

## Conclusions

SMA has multidimensional medical, psychological, social and practical implications for adults. Despite an established evidence base for clinical care of SMA in children, this review highlights the significant knowledge gaps in best practice management, patterns of treatment response, and disease progression in adults with SMA. From existing research, adults with SMA experience limitations in mobility and daily activities associated with a gradual deterioration in motor function, alongside emotional difficulties, fatigue and a perceived lack of societal support, however there was no evidence regarding effective interventions. Psychological well-being was important for adults with SMA, however there was limited data regarding prevalence and burden of mental health with no high quality evidence regarding effective interventions. Identifying gaps in knowledge is essential to guide future clinical research, best practice care, and advance health policy with the ultimate aim of reducing the burden associated with adult SMA.

## Data Availability

The datasets used and/or analysed during the current study are available from the corresponding author on reasonable request.

## Abbreviations

6MWT: Six-minute walk test
EK: Egen Klassifikation;
FVC: Forced vital capacity
GMFM: Gross motor function measure
HFMS: Hammersmith functional motor scale
HFMSE: Hammersmith functional motor scale expanded
MFM: Motor function measure
MI-E: Mechanical insufflation-exsufflation
MMT: Manual muscle testing
MRC: Medical Research Council
PRISMA: Preferred Reporting Items for Systematic reviews and Meta-Analyses
QoL: Quality of life
RULM: Revised Upper Limn Module
SMA: Spinal muscular atrophy
TMS: Total muscle score
ULM: Upper limb module
VO_2_Max: Maximal oxygen uptake
